# Health-related quality of life in nondialysis CKD patients: a comprehensive description of five-year trajectories among the CKD–REIN cohort

**DOI:** 10.1186/s12882-025-04702-2

**Published:** 2026-01-07

**Authors:** Moustapha Faye, Lisa Le Gall, Aghilès Hamroun, Lucile Montalescot, Karen Leffondre, Natalia Alencar de Pinho, Bénédicte Stengel, Adama Faye, Luc Frimat, Abdou Omorou

**Affiliations:** 1 CHRU-Nancy, INSERM, Université de Lorraine, CIC, Épidémiologie Clinique, INSPIIRE, Nancy, F-54000 France; 2 Service de Néphrologie – CHU Aristide Le Dantec, Université Cheikh Anta Diop, Dakar-Fann, BP 5005 Sénégal; 3 University Bordeaux, INSERM, BPH, U1219, CIC 1401, Bordeaux, F-33000 France; 4 Public Health - Epidemiology, Nephrology Department, Lille University Hospital Center, Lille University, Lille, France; 5 UMR1167 RID-AGE, Pasteur Institute of Lille, Inserm, Lille, France; 6 Laboratoire de Psychopathologie et Processus de Santé, Université Paris-Cité, Boulogne-Billancourt, France; 7 Centre for Research in Epidemiology and Population Health (CESP), Paris-Saclay University, Inserm, Clinical Epidemiology team, Villejuif, 94800 France; 8 Institut Santé et Développement (ISED), Université Cheikh Anta Diop, Dakar, Sénégal; 9 Service de Néphrologie, CHRU de Nancy, Vandœuvre-lès-Nancy, 54500 France

**Keywords:** Chronic kidney disease, Clinical epidemiology, Cohort studies, Mental component summary, Physical component summary

## Abstract

**Background:**

Few studies have analyzed the quality-of-life trajectories of CKD patients not receiving kidney replacement therapy, and the results are inconsistent. This study aimed to identify subgroups of long-term trajectories of the physical (PCS) and mental components summary (MCS) of the KDQOL-36 in patients with CKD stages 3–5 and to describe their associations with patient characteristics.

**Methods:**

We used a joint latent class–mixed model to identify the PCS and MCS trajectories of 2716 patients with CKD stages 3–5 enrolled in the CKD–Renal Epidemiology and Information Network (CKD–REIN) cohort study. Quality-of-life was assessed annually using the Kidney Disease Quality-of-life-36. All the participants had scores for at least one-time point.

**Results:**

During a median follow-up of 5.56 (4.77–6.16) years, 664 participants started KRT, and 465 died before KRT. We identified three profiles of PCS: a “High and declining PCS trajectory” which included 5.89% of patients, characterized by a higher initial score and a decline of more than 10 points over three years; a “High and stable PCS trajectory” in 50.96%, characterized by a higher initial score that remained stable; and a “Low and stable PCS trajectory” in 43.15%, characterized by a lower initial score that remained stable. For MCS, we identified a single, stable mean trajectory over time. A decline in eGFR was faster in participants with a “High and declining PCS trajectory” (-4.30 mL/min per years). Patients in the high and stable trajectory had a more favorable clinical and biological profile at baseline. The evolution of the specific dimensions of CKD within each PCS trajectory followed a pattern similar to that of the PCS itself.

**Conclusions:**

The study highlights substantial heterogeneity in PCS evolution in patients with CKD, which contrasts with the stability of that for MCS.

**Supplementary information:**

The online version contains supplementary material available at 10.1186/s12882-025-04702-2.

## Introduction

Health-related Quality-of-life (HRQOL), a multidimensional concept, is an important clinical and economic outcome for patients, health care providers, researchers and payers. [1, 2] Patients with late-stage chronic kidney disease (CKD), comorbidities and treatments such as dialysis experience significant lifestyle changes that affect their HRQOL. [3–5] The more advanced the CKD is, the more HRQOL is impaired. [2, 4, 6] This association between CKD and HRQOL has been demonstrated mainly by cross-sectional studies. [2, 4–6] Currently, data on the longitudinal trajectory of HRQOL in CKD patients are lacking. A few longitudinal studies have investigated HRQOL evolution in chronic dialysis patients. [7–12] Longitudinal studies [2, 13–15] of physical (PCS) and mental (MCS) HRQOL domains in CKD patients not receiving kidney replacement therapy (KRT) have inconsistent results, possibly due to differences in study design, population characteristics (age, CKD severity at inclusion), and limitations in the statistical methods used. Few studies have sought to classify patients according to the evolving profile of their Quality-of-life. [2, 13]

We hypothesized that (i) we could identify subgroups of patients with different evolutions of HRQOL over time and that (ii) these subgroups would exhibit different clinical and biological characteristics. For this purpose, we used longitudinal data from the French Chronic Kidney Disease-Renal Epidemiology and Information Network (CKD–REIN) cohort study to identify subgroups of long-term trajectories of the PCS and MCS of the KDQOL-36 in patients with CKD stages 3–5 and to describe their associations with patient characteristics at baseline, eGFR, KDQOL symptoms, burden, and effect trajectories and outcomes (KRT and death before KRT).

## Materials and methods

### Study design, population, and setting

The study design and patient profile have been described in detail by Stengel et al. [16] In summary, the CKD–REIN study is a prospective cohort study conducted in 40 nephrology clinics that are nationally representative geographically and in terms of facility legal status (public, private not for profit, and private for profit). Between July 2013 and March 2016, a total of 3033 adults over 18 years of age with a proven CKD diagnosis, estimated glomerular filtration rate (eGFR) < 60 mL/min/1.73 m^2^, and no KRT were included in the cohort during a routine visit to their nephrologist. The participants were followed by clinical research associates until KRT initiation (dialysis or transplantation), death or loss to follow-up. In the present analyses, all patients with at least one available score during the study period were included. This study was reported using the Strengthening the Reporting of OBservational studies in Epidemiology (STROBE) checklist [17] (Supplemental Table S1).

### Data collection

The information collected at baseline included sociodemographic data (age, sex, education, marital status, monthly income), clinical data (diabetes mellitus, cardiovascular comorbidities), smoking status, body mass index (BMI; in kg/m^2^), and biological data (hemoglobin, serum creatinine, and albumin levels; eGFR using the CKD-EPI 2009 equation [18] (eGFR); the urinary albumin-to-creatinine ratio (ACR)). Definitions of the operational variables are reported in Supplemental Table S2.

Upon enrollment and each year thereafter, all study participants were asked to complete a self-administered patient questionnaire including the validated French version of the KDQOL. [19, 20] We computed five-dimensional scores, such as burden, effect, and symptom scores, as well as PCS and MCS. [20] Higher scores indicate better HRQOL.

The judgment criteria were the PCS and the MCS scores from the KDQOL-36 questionnaire. [20] The KDQOL™ scoring program was used for scoring five-dimensional scores developed by RAND Health Care [21].

Kidney failure with replacement therapy (KFRT) events were identified from medical records, patient interviews, or by linkage with the national REIN (Renal Epidemiology and Information Network) registry. [22] Deaths were ascertained from death certificates, hospital records, reports by family members, and linkages with the national death registry.

### Statistical analyses

#### Descriptive analysis

The baseline characteristics of the patients are described as numbers (percentages) or means (±SDs) and/or medians (interquartile ranges [IQRs]). The included and non-included patients were compared by standard statistical tests (Student’s *t* test or the Mann‒Whitney test for continuous variables and the chi‒square or Fisher test for categorical variables, depending on sample size).

### Identification of subgroups of HRQOL trajectories over time

We used a Joint Latent Class Mixed Model (JLCMM) to identify subgroups of physical and MCS trajectories over time. The time of origin was the date of inclusion in the cohort, and the administrative censoring date was December 31, 2020.

The JLCMM is an extension of the latent class mixed model (LCMM) to account for potential informative dropouts such as KFRT or death before. [23] It allows for the consideration of patients with only one measure to reduce selection bias. [24, 25] The JLCMM consists of three joint submodels: a multinomial logistic regression model for estimating the probability of each patient belonging to each latent class, a class-specific mixed model for modeling trajectories over time, and two cause-specific proportional hazards models to account for informative dropout (missing not at random). The two events (death and KRT) were modeled in a competing risk setting. None of the submodels introduced patient characteristics because our aim was to identify latent classes independent of these characteristics. Separate JLCMMs were constructed to identify PCS and MCS trajectories.

In the preliminary analysis, because the longitudinal PCS score was not normally distributed, we tested different transformations, and the natural spline with 7 interior knots at the quantiles best fit the data according to the Akaike information criterion (AIC). For MCS score, a linear link function was used because it was normally distributed. We also tested different functions of time (in years) and chose a natural spline with one interior knot at the median for PCS and a linear function for MCS with an unstructured variance‒covariance matrix for the random effects. For the two cause-specific proportional hazards models, we also tried different baseline hazard functions and selected the Weibull distribution for the MCS and PCS. All the details of the modeling choices are shown in Supplemental Text 1.

To choose the optimal number of latent classes, we constructed several JLCMMs for each component with one to five classes each and selected the final model on the basis of trade-offs among [Supplemental Tables S3 and S4] 1) data fit (according to the Bayesian information criterion), 2) discrimination between classes (according to entropy (the higher the entropy, the better the discrimination) and the posteriori classification table [Supplemental Table S5]), and 3) the clinical relevance of the results. [26, 27] We excluded models with small class sizes (less than 5% of the total sample).

### Patient characteristics in each subgroup of trajectories

Exploratory, secondary analysis of factors associated with each PCS trajectory was based in complete cases (thus relying on a missing completely at random assumption). The percentages of missing data for each covariate were mostly below 10%, and are reported in the footnote of Table 1. To compare baseline characteristics across the different HRQOL trajectory profiles, we used secondary multinomial model for external class predictor fitted by maximum likelihood method with total parameter variance estimated using parametric bootstrap and correction for primary model uncertainty (*ExterVar* function of the lcmm R package) [28]. To describe the evolution over time of the eGFR, symptoms, burden and effects of kidney disease in each class, we used two-stage linear mixed models [28] to account for the primary model uncertainty classification.

**Table 1:** Baseline characteristics of patients according to their subsequent PCS trajectory phenotype (*n* = 2716)

Characteristics	Overall	PCS profiles		
		“High and declining PCS trajectory”	“Low and stable PCS trajectory”	“High and stable PCS trajectory”
n (%)	2716 (100)	160 (5.89)	1172 (43.15)	1384 (50.96)
Age (in years)	66.89 ± 12.59	59.62 ± 15.95	70.04 ± 11.59	65.07 ± 12.27
Age group (in years)				
18–44	172 (6.3)	30 (19)	42 (3.6)	100 (7.2)
45–64	772 (28)	55 (34)	267 (23)	450 (33)
65–74	962 (35)	46 (29)	398 (34)	518 (37)
≥75	810 (30)	29 (18)	465 (40)	316 (23)
Male sex	1792 (66)	118 (74)	737 (63)	937 (68)
Currently married	1658 (61)	96 (60)	699 (60)	863 (62)
Education level (in years)				
< 9	370 (14)	12 (7.5)	206 (18)	152 (11)
9–12	1323 (49)	71 (45)	614 (53)	638 (47)
> 12	992 (37)	76 (48)	338 (29)	578 (42)
Monthly income (in euros)				
< 1500	496 (46)	20 (13)	269 (23)	207 (15)
1500–4200	1254 (46)	79 (49)	506 (43)	669 (48)
> 4200	299 (11)	31 (19)	86 (7.3)	182 (13)
BMI (kg/m^2^)	28.65 ± 5.78	26.76 ± 4.72	29.83 ± 6.32	27.87 ± 5.21
Body weight status				
Underweight	40 (1.5)	4 (2.5)	15 (1.3)	21 (1.5)
Healthy weight	716 (26)	54 (34)	257 (22)	405 (29)
Overweight	980 (36)	68 (43)	373 (32)	539 (39)
Obesity	927 (34)	32 (20)	498 (42)	397 (29)
Primary kidney disease				
Glomerulonephritis	479 (18)	50 (31)	172 (15)	257 (19)
Diabetes mellitus	533 (20)	15 (9.4)	307 (26)	211 (15)
Interstitial nephropathy	344 (13)	19 (12)	131 (11)	194 (14)
Kidney vascular disease	757 (28)	25 (16)	332 (28)	400 (29)
ADPKD	150 (5.5)	30 (19)	52 (4.4)	68 (4.9)
Others	291 (11)	13 (8.1)	106 (9.0)	172 (12)
Diabetes mellitus	1141 (42)	40 (25)	616 (53)	485 (35)
Cardiovascular history	1414 (52)	66 (41)	764 (65)	584 (42)
Heart failure	337 (12)	8 (5.0)	226 (19)	103 (7.4)
Charlson Comorbidity Index ≥ 5	2066 (76)	83 (52)	1022 (87)	961 (69)
Cancer	571 (22)	20 (13)	277 (24)	274 (21)
PCS*	41.57 ± 10.01	51.55 ± 4.79	34.50 ± 8.33	46.35 ± 7.65
MCS*	47.69 ± 7.23	46.84 ± 7.71	47.52 ± 7.45	47.94 ± 6.98
Burden score*	74.83 ± 23.73	79.63 ± 19.13	66.17 ± 25.63	81.61 ± 19.88
Effect score*	81.70 ± 17.52	86.48 ± 11.59	74.92 ± 19.30	86.92 ± 14.23
Symptoms score*	75.50 ± 16.34	84.91 ± 10.88	68.36 ± 17.20	80.47 ± 13.48
Depression score (CES-D)*	25.02 ± 17.09	17.78 ± 12.95	30.32 ± 17.73	21.39 ± 15.65
Physical activity (GPAQ)				
Intense	679 (28)	53 (35)	195 (19)	431 (34)
Moderate	612 (25)	42 (28)	222 (21)	348 (28)
Low	1170 (48)	55 (37)	635 (60)	480 (38)
eGFR (mL/min/1.73 m^2^)	33.21 ± 12.14	24.49 ± 8.92	29.32 ± 11.29	37.51 ± 11.50
eGFR < 30 mL/min/1.73 m^2^)	1201 (44)	127 (79)	680 (58)	394 (28)
Urinary albumin‒creatinine ratio (mg/g)				
< 30	697 (26)	13 (8.1)	218 (19)	466 (34)
30 to 299	782 (29)	37 (23)	332 (28)	413 (30)
≥300	1002 (37)	101 (63)	518 (44)	383 (28)
Serum albumin < 40 g/L	205 (7.5)	15 (9.4)	127 (11)	63 (4.6)
Anemia**	1005 (37)	75 (47)	558 (48)	372 (27)
Systolic blood pressure (mmHg)	142.26 ± 20.47	143.02 ± 20.03	144.55 ± 21.25	140.23 ± 19.63
Diastolic blood pressure (mmHg)	78.19 ± 12.10	81.84 ± 12.42	77.05 ± 12.36	78.72 ± 11.73
Transplant list registration	391 (14)	111 (69)	180 (15)	100 (7.2)
Reported ever attending an education session	591 (23)	55 (35)	273 (25)	263 (20)

All data are presented as n (%) or mean ± SD

Missing data: no missing data: age, sex, eGFR; ≤ 5% of missing data: education, BMI, diabetes mellitus, cardiovascular history, cancer, Charlson Comorbidity Index, symptoms score, calcium, potassium, anemia, systolic and diastolic blood pressure; between 5 and 10% of missing data: burden score, effect of the kidney disease score, depression score, marital status, ACR, monthly income, and physical activity; ≥ 10% of missing data: PCS, MCS, and serum albumin

Abbreviations: BMI, body mass index; eGFR, estimated glomerular filtration rate; ADPKD, Autosomal dominant polycystic kidney disease; MCS, mental component summary; PCS, physical component summary; CKD, chronic kidney disease; GPAQ, Global Physical Activity Questionnaire; CES-D, Center for Epidemiologic Studies Depression Scale

^*^ A higher score indicates the presence of more depression or best quality of life (burden, effect, symptoms)

^**^ Hemoglobin level < 12.0 g/dL in women and < 13.0 g/dL in men

### Sensitivity analyses

In the sensitivity analysis, we used the *lcmm* function from the lcmm package to identify PCS and MCS trajectories over time. This function does not take events into account, even if they are potentially informative dropout.

All analyses were conducted with R version 4.5.0 software via the R package LCMM.

## Results

### Patient characteristics

**Fig. 1:**
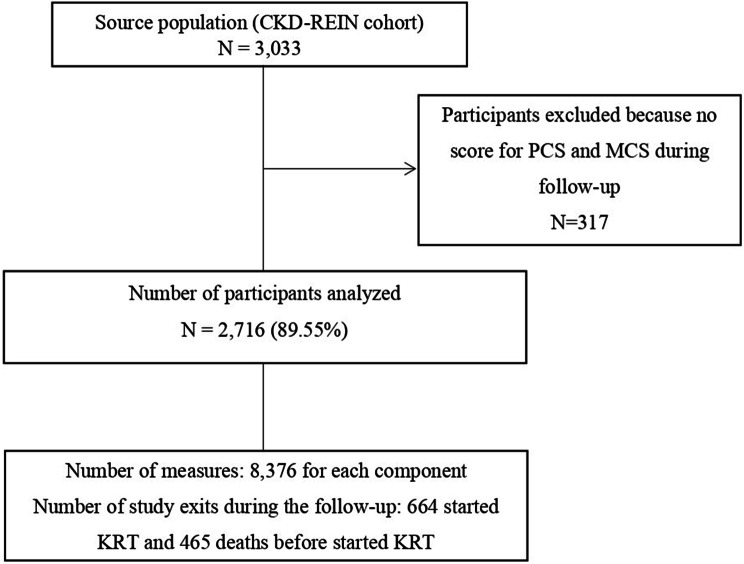
Flow chart of the study population. Chronic kidney disease-renal epidemiology and information Network (CKD–REIN) cohort

In this study, we analyzed data from 2716 patients who provided at least one score for the KDQOL-36 PCS and MCS during the follow-up period, from the inclusion date to December 31, 2020. These patients reported a total of 8376 measures of the component summaries as shown in Fig. 1. During follow-up, 25% (*n* = 685), 21% (*n* = 557), 17% (*n* = 466), 18% (*n* = 483) and 19% (*n* = 525) of the participants had completed 5, 4, 3, 2 and 1 KDQOL-36 questionnaires, respectively. The baseline characteristics of the 2716 participants are shown in Table 1. The mean (± SD) age was 66.89 ± 12.59 years, and 66% were men. The mean eGFR was 33.21 ± 12.14 ml/min/1.73 m^2^ and 44% of the participants had an eGFR < 30 mL/min/1.73 m^2^. The mean PCS and MCS scores were 41.57 ± 10.01 and 47.69 ± 7.23, respectively. Compared with the included participants, the 317 excluded participants more often had diabetes, obesity, anemia, hypoalbuminemia, and depression and had lower eGFRs and lower symptoms, effect and burden scores. These patients also experienced more events (death or KRT) [Supplemental Table S6]. During a median follow-up of 5.56 (4.77–6.16) years, 664 participants started KRT, and 465 died before KRT.

### Profiles of physical HRQOL trajectories

**Fig. 2:**
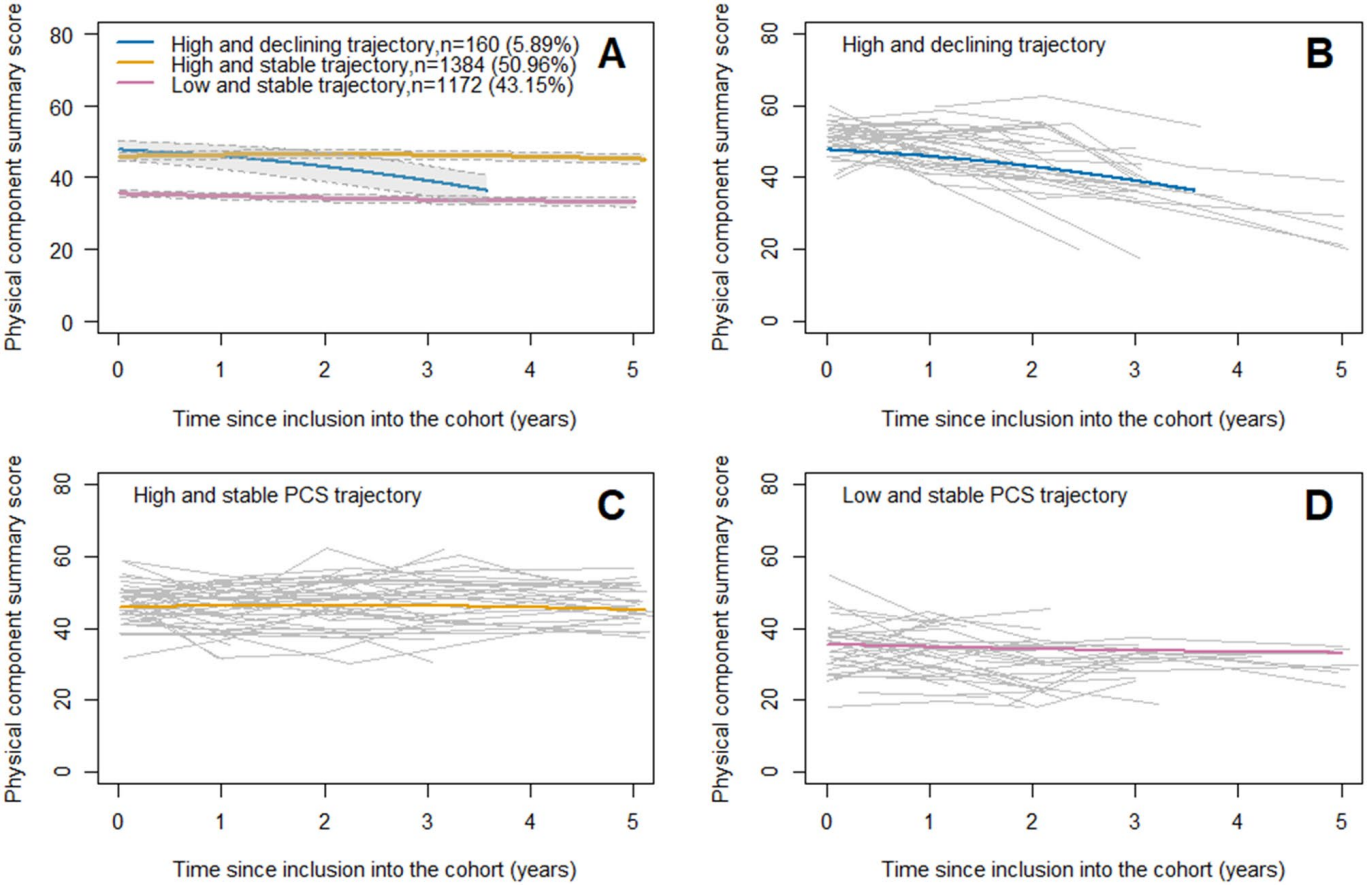
Predicted mean trajectories of PCS. The panel **A** shows the predicted mean trajectories (solid line) of PCS in the three identified latent classes and their confidence intervals (dotted line). The lower the PCS score is, the worse the physical component of HRQOL. The panels **B** shows the observed individual trajectories of 50 randomly selected participants a posterior classified in the “High and declining trajectory”. The panel **C** in the “High and stable trajectory” and the panel **D** in the “low and stable trajectory”. Chronic kidney disease-renal Epidemiology and information Network (CKD–REIN) cohort

The best-fit JLCMM had three profiles as illustrated in Fig. 2A and Supplemental Table S3. Model discrimination is low because the entropy was 0.6. [29] The mean posterior probability in each class was greater than 0.70, except for class 1 [Supplemental Table S5].

The “High and declining PCS trajectory” (*n* = 160; 5.89%) was characterized by a higher initial level of PCS score than that observed in the low-stable group (mean PCS score: 51.55 ± 4.79), with a rapid decline of more than 10 points over three years of follow-up. The “High and stable PCS trajectory” (*n* = 1384; 50.96%) was characterized by a higher initial level of PCS score than that observed in the low-stable group (mean PCS score: 46.35 ± 7.65) that remained stable over time. The “low and stable PCS trajectory” (*n* = 1172; 43.15%) was characterized by a lower initial level of PCS score (mean PCS score: 34.50 ± 8.33) that remained stable over time. The observed individual PCS trajectories of 50 randomly selected participants some posteriori classified in each class are displayed in Fig. 2B, 2C and 2D.

### Profile of mental HRQOL trajectories

**Fig. 3:**
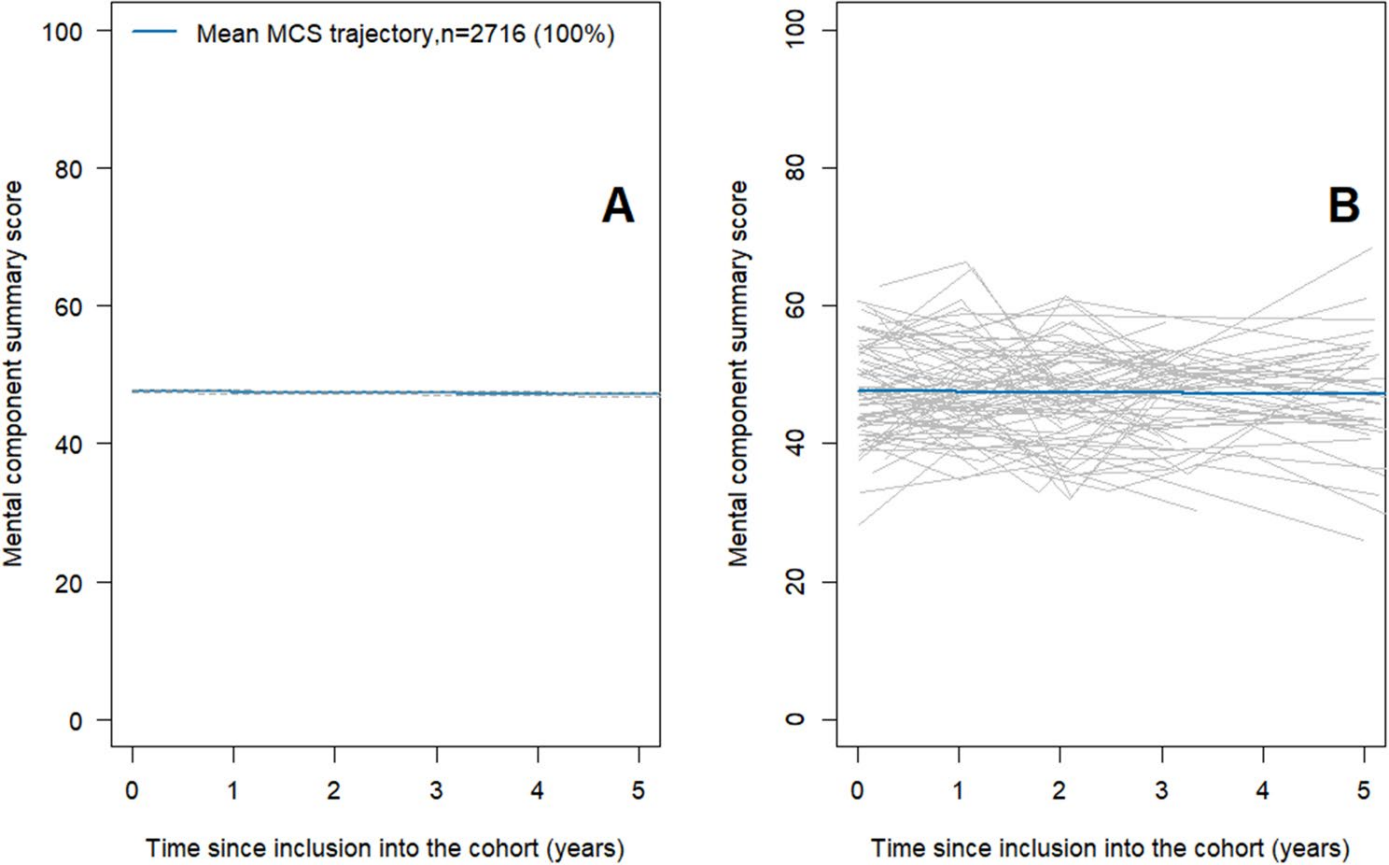
Predicted mean trajectory of MCS. The panel **A** shows the predicted mean stable trajectory (solid line) of MCS in the single identified latent class and its confidence interval (dotted line). The panel **B** shows the observed individual trajectories of 100 randomly selected participants. The lower the MCS score is, the worse the mental component of HRQOL. Chronic kidney disease-renal Epidemiology and information Network (CKD–REIN) cohort

The best fitted JLCMM identified a single profile characterized by a stable MCS score over time, as illustrated in Fig. 3 and Supplemental Table S4.

### Baseline characteristics of patients in each profile of physical HRQOL trajectories

Compared with patients included in the “low and stable PCS trajectory”, those with a “high and declining PCS trajectory” were younger. These patients had lower eGFR and lower body mass index (BMI) and less depressed. Those with a “high and stable PCS trajectory” were younger; less depressed; and less likely to have diabetes, anemia, hypoalbuminemia and comorbidities (Table 2).

**Table 2:** Baseline characteristics of patients according to PCS trajectories. The low and stable trajectory was used as the reference. The analysis was conducted on a sample of 2009 patients with complete data

	Low and stable PCS trajectory = reference			
	High and declining PCS trajectory		High and stable PCS trajectory	
	aOR (95% CI)	p-value	aOR (95% CI)	p-value
Age (in years)	0.888 (0.851 - 0.927)	< 0.001	0.936 (0.904 - 0.968)	< 0.001
Sex (men/women)	1.766 (0.822 - 3.793)	0.145	1.273 (0.757 - 2.142)	0.362
BMI (in kg/m^2^)	0.901 (0.842 - 0.964)	0.002	0.892 (0.853 - 0.932)	< 0.001
eGFR (in ml/min/1.73 m^2^)	0.881 (0.813 - 0.956)	0.002	1.097 (1.068 - 1.128)	< 0.001
Anemia (yes/no)	0.765 (0.371 - 1.579)	0.469	0.399 (0.255 - 0.624)	< 0.001
Diabetes mellitus (yes/no)	0.437 (0.194 - 0.983)	0.045	0.391 (0.242 - 0.633)	< 0.001
Charlson Index (≥5/ < 5)	0.433 (0.138 - 1.361)	0.152	0.387 (0.165 - 0.907)	0.029
Depression score (CES-D)	0.928 (0.902 - 0.955)	< 0.001	0.936 (0.920 - 0.953)	< 0.001
Serum albumin < 35 g/L (yes/no)	0.591 (0.197 - 1.777)	0.349	0.191 (0.078 - 0.469)	< 0.001
Literacy (yes/no)	2.441 (0.845 - 7.050)	0.099	1.492 (0.854 - 2.605)	0.160
Living alone (yes/no)	1.193 (0.498 - 2.861)	0.692	1.232 (0.739 - 2.052)	0.423
Number of nephrology visit	1.118 (0.993 - 1.260)	0.066	1.210 (1.115 - 1.313)	< 0.001

*Abbreviations:* PCS, Physical Component Summary; aOR, adjusted Odds ratio; CI, confidence interval; BMI, Body mass Index; eGFR, estimated glomerular filtration rate. The depression score was assessed using the Center for Epidemiologic Studies Depression Scale (CES-D) questionnaire. with higher scores indicating greater depressive symptoms. Anemia was defined as a hemoglobin level < 13 g/dL in men and < 12 g/dL in women

This analysis was performed using a secondary multinomial model for the external class predictor, with the total parameter variance estimated by parametric bootstrap, including a correction accounting for the uncertainty of the primary model

### Longitudinal characteristics of patients in each subgroup of physical HRQOL trajectories

**Fig. 4:**
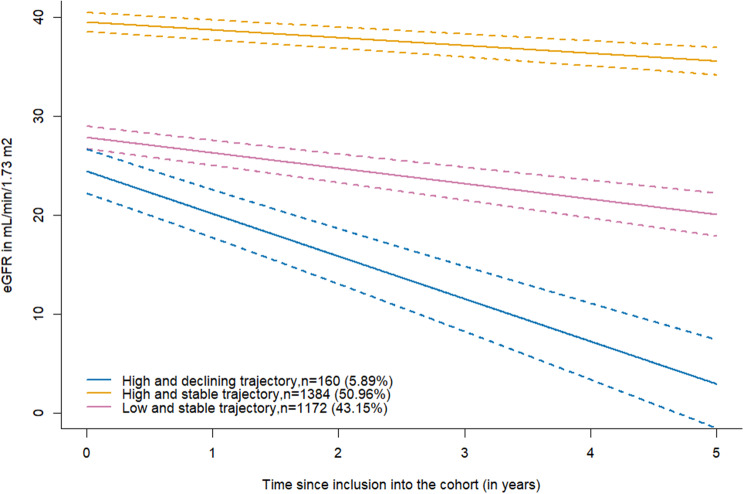
eGFR evolution (solid line) according to the three identified trajectories of PCS and their confidence intervals (dotted line). Chronic kidney disease-renal Epidemiology and information Network (CKD–REIN) cohort

**Fig. 5:**
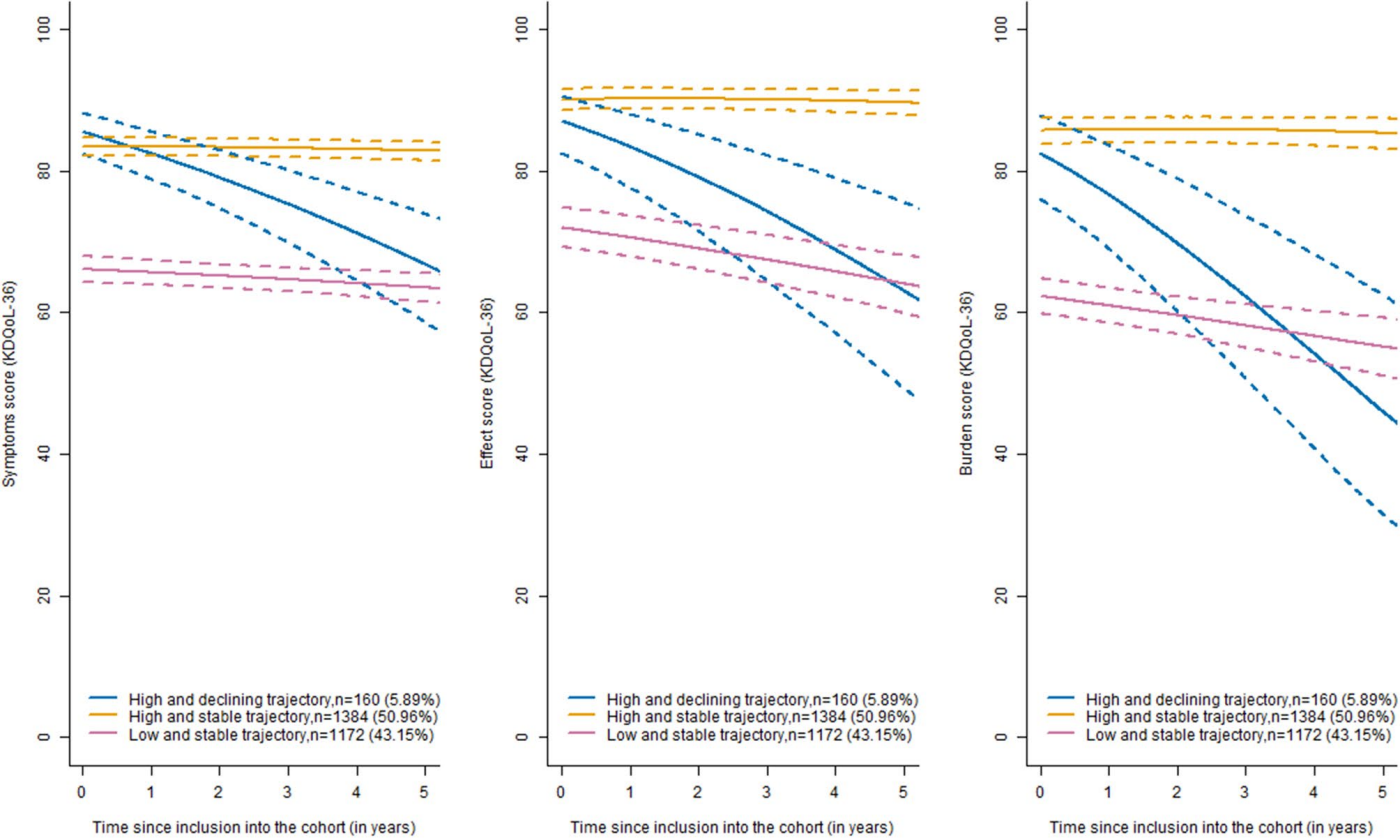
Symptom (left). effect (medium) and burden (right) score evolution (solid line) by three identified phenotypes of PCS and their confidence intervals (dotted line). Chronic kidney disease-renal Epidemiology and information Network (CKD–REIN) cohort

During follow-up, dialysis was initiated more frequently among patients in the “High and declining trajectory” (70%), followed by those in the “Low and stable trajectory” (34%) and the “High and stable trajectory” (5%). Kidney transplantation occurred in 23% of patients in the “High and declining trajectory”, 3% in the “Low and stable trajectory”, and only a very small proportion in the “High and stable trajectory” (1%). Death occurred in 37% of patients in the “Low and stable trajectory”, 5% in the “High and declining trajectory”, and 2% in the “High and stable trajectory”. Patients with a “High and declining PCS trajectory” had a low initial level of eGFR (24.4 mL/min/1.73 m^2^) with a fast decline over time (−4.30 mL/min/1.73 m^2^ per year; *p* < 0.001) and a high initial level of symptoms, burden and effect (the higher the score was, the fewer symptoms and effects and lower burden) with a rapid decline over time. Patients belonging to the “Low and stable PCS trajectory” had intermediate eGFR (27.82 mL/min/1.73 m^2^) with a small decline over time (−1.56 mL/min/1.73 m^2^ per year; *p* < 0.001), a low initial level of symptoms, burden and effects that remained stable over time and a higher probability of death before KFRT [Supplemental Figure S1]. Participants belonging to the “High and stable PCS trajectory” had better initial eGFR (39.46 mL/min/1.73 m^2^), which a very small decline over time (−0.79 mL/min/1.73 m^2^ per year; *p* < 0.001). The evolution of the eGFR according to the PCS trajectories is shown in Fig. 4. The symptoms, burden, and effect evolution by PCS trajectories are shown in Fig. 5.

### Sensitivity analyses

In sensitivity analyses, the best model had two PCS trajectories and one MCS trajectory (Supplemental Table S7 and S8). The “high and stable PCS trajectory” included 202 (7.44%) participants and the “Low and stable PCS trajectory” included 2514 (92.56%) participants (Supplemental Figure S2).

## Discussion

This longitudinal study of HRQOL in patients with CKD not receiving KRT made it possible to identify three profiles for PCS trajectories. A majority of patients had a high and stable PCS trajectory, indicating favorable physical health evolution, but more than one-third had a stable low or rapidly declining trajectory reflecting poor physical health. In contrast, there did not appear to be any heterogeneity in the mental health trajectory. The model simply assumes that there are no subgroups of MCS evolution and that all individuals follow, more or less, the same trajectory over time.

The names of the PCS trajectories were chosen solely for descriptive and comparative purposes among the three identified trajectories. We therefore used the term “high PCS” to indicate the subgroup with relatively better physical health status within our cohort. The low and stable trajectory had a mean initial score 11.85 points lower than the high and stable trajectory and 17.05 points lower than the high and declining trajectory. Moreover, in a study based on the CKD-REIN cohort, Legrand et al. [4] reported a mean PCS score of 50.2 ± 9.1 in the general French population, which is very close to the mean scores observed in the first two PCS trajectories of our study. In addition, this study reported that the mean PCS score was 42.6 ± 9.9 among patients with an eGFR > 30 mL/min. In contrast, the low and stable trajectory showed a much lower mean score (34.40) compared with these populations.

This study is, to our knowledge, the first to examine HRQOL trajectories in CKD patients not receiving KRT using the JLCMM model. [23] This model takes into account three major aspects: the “missing not at random” nature of longitudinal data in HRQOL assessments, the risk of selection bias when restricting analysis to patients with repeated measurements, and the intraindividual variability of measurements. The optimal number of classes was chosen based on trade-offs among fit indices, classification quality, and clinical relevance. Each individual was assigned to a trajectory according to their posterior probability of class membership. The higher this probability, the lower the individual’s classification uncertainty. Although there is no agreed upon cutoff criterion for entropy, value closer to 1, indicate better classification. Despite being systematically rerun several times and using of grid search to avoid convergence to a local maximum likelihood, the model yielded an entropy of 0.6 (borderline acceptable) [30] and average posterior probabilities between 0.67 to 0.82 (Supplemental Table S5). However, clinical relevance, in addition to parsimony, particularly supported the choice of the three-class model, while acknowledging some classification uncertainty, especially in class 1.

Our results are consistent with earlier findings from Legrand et al. [4], who reported a significant gradual decline in PCS with worsening CKD stages in contrast to MCS. Several other cross-sectional analyses revealed that the lower the eGFR was, the lower the PCS, but not the MCS. [13, 31, 32] Several longitudinal studies have examined the mean HRQOL over time and reported inconsistent results regarding the course of HRQOL. [2, 15, 33–35] These inconsistent results might be due to differences in study design or baseline participant characteristics (age, eGFR and comorbidities); moreover, examining the mean HRQOL over time can mask individual variations in the course of HRQOL [14] and does not account for correlations between measurements. Other studies have identified different HRQOL trajectories over time using clustering or latent class analyses and reported discordant results. In the prospective PREdialysis PAtient REcord-2 (PREPARE-2) study, the SF-36 was completed every six months (396 participants with a mean age of 64.4 ± 14.0 years and a mean eGFR of 16.8 ± 6.1 ml/min/1.73 m^2^), Meuleman et al. [14], used latent class growth models, identified three PCS trajectories (low-stable [34.1%], medium-declining [32.5%], and high-increasing [33.4%]) and two MCS trajectories (low-stable [38.7%] and high-stable [61.3%]). Thus, a large proportion of the PREPARE-2 sample [14] had an unfavorable (i.e., stable low or declining) PCS trajectory, which is consistent with our results. In a population of patients in the Chronic Renal Insufficiency Cohort (CRIC) study who were 10 years younger than our participants and had a baseline eGFR of 44 ml/min/1.73 m^2^, which was higher than that in our study, Grams et al. [13] also found that PCS and MCS were relatively stable over time among three prespecified groups, as identified by an unsupervised clustering approach, but differed in the baseline PCS and MCS scores. The latent class growth models take into account the individual variation in the course of HRQOL but not the correlations between measurements and potential informative dropout due to KFRT or death before KFRT, unlike the JLCMM.

We also found that participants belonging to the “Low and stable PCS trajectory” were older, had a slow decline in the eGFR trajectory over time, and more comorbidities. Participants belonging to the “High and declining PCS trajectory” were younger and had a very fast decline in the eGFR trajectory over time. Thus, it is likely that changes in PCS score are driven primarily by CKD progression in younger patients, whereas in older adults they are driven mainly by comorbidities. Grams et al. [13] also reported different risks of death, cardiovascular disease and kidney failure according to prespecified subgroups of MCS and PCS. In addition, patients in the “High and stable trajectory” had a more favorable clinical and biological profile at baseline. Among older adults ≥ 65 years, Chesnaye et al. reported that men experienced a more rapid decline in HRQOL over time than women, which contrasts with our findings. [35] Additionally, the course of symptoms, effects and burden of kidney disease was superimposable on the evolution of PCS. Among patients in the “High and declining trajectory”, the baseline PCS score was comparable to that of the French general population. [4]

The stability of the mean MCS trajectory over time may be linked to a problem of KDQOL responsiveness in CKD patients. [36] In the CKD–REIN cohort study, Legrand et al. [4] reported that crude MCS scores were similar between the general population and all kidney disease patient subgroups except those receiving dialysis. This stability over time can also be explained by the ability to cope mentally and emotionally with a crisis or to return to precrisis status quickly (psychological resilience). [37] Another explanation might be that patients do not identify any stressors linked to their illness, both explaining their high HRQOL and its stability. Cognitive avoidance, such as the denial of the disease, involves a variety of coping strategies. [38] Montalescot et al. [39] found that non-dialysis CKD patients in the CKD–REIN cohort tended to avoid thinking about their disease and report little psychological impact of their disease, even stating that their lives are relatively normal and that they do not feel ill.

Our findings have many clinical implications. The identification of distinct trajectories offers the possibility to individualize follow-up, adapt therapeutic interventions to patients’ needs, improve the early identification of patients at risk of clinical deterioration and poor quality of life, and help optimize the use of healthcare resources. Patient-centeredness, including shared decision-making, has emerged as a clinical dimension for maximizing the quality of care on the basis of patient-valued outcomes. [40] Our results and much evidence from the nephrological literature and that of other chronic diseases suggest that regular patient-reported outcome measure (PROM) use with clinician follow-up is possible and can enhance patient‒clinician communication [41, 42], facilitate the reporting of serious adverse events [43], and help nephrologists teams develop strategies to improve CKD HRQOL. [44] Regular PROM use can also be used to integrate routinely collected clinical and laboratory data (big PRO data) with several opportunities in patient care, population health management, and research. [45] Routine and active assessment of HRQOL over time will allow for better follow-up and management of HRQOL and better adherence of CKD patients to treatment. [46] For these profits, the Kidney Disease Improving Global Outcomes (KDIGO) controversies conference in trends and perspectives for improving the quality of CKD care [40] list several key considerations, including PROM adaptation and validation, burden of measurement, potential patient-level barriers, potential system/clinician-level barriers and the role of technology and availability of electronic tools. Unfavorable evolution of the PCS is associated with rapid decreases in kidney function and symptom, burden and effect scores. Several questions remain to be answered in relation to the active HRQOL monitoring in clinical practice: what is the alert threshold? How often should this monitoring be carried out? What is the best PROM to use? what is the cost‒benefit ratio? What are the management strategies for HRQOL decline? Further investigations are needed to answer these questions. Clinicians, in collaboration with others involved in CKD management, must above all put in place strategies for monitoring, alerting and managing physical HRQOL. Several support strategies are available: education [47], adapted physical activities, challenge misconceptions, and the development of action plans. [14, 48]

The sensitivity analysis shows that accounting for events in the trajectory modeling appears to have an effect for PCS trajectories but not for MCS. The LCMM model identified two stable PCS trajectories, in contrast to the JLCMM model, while MCS remains homogeneous and stable. Thus, with or without accounting for potentially informative events, the evolution of PCS is heterogeneous yet stable in the majority of patients, whereas that of MCS is homogeneous.

The main strengths of this study are the large sample size of participants included, with various CKD etiologies and repeated measurements of PROMs such as the KDQOL-36 questionnaire over a 5-year follow-up. This cohort included patients recruited from a representative sample of nephrology outpatient clinics in France. Clinical outcomes such as PRO were rigorously collected, with high response rates ranging from 89% at baseline to 67% at the 5-years follow-up. Over 45% of the cohort participants had at least 4 responses. JLCMM are useful for identifying different subgroup trajectories over time. [23, 28] However, this study also has limitations. There is potential selection bias because the 317 non-included participants had a worse baseline clinical profile and experienced more adverse events (KFRT or death before) than those included. Therefore, we may have underestimated the proportion of patients with unfavorable PCS profiles. The detection of HRQOL changes over time is closely related to the psychometric properties of the KDQOL-36, including its responsiveness (sensitivity to change). However, the KDQOL-36 remains the most widely used PROM in the assessment of HRQOL, has the best psychometric properties and allows for comparisons between studies. [36] The issue of a response shift is important to consider when interpreting self-reported changes. The *response* reflects the fact that patients make an assessment, judgment, or rating of a health state, and a *shift* implies change, more specifically, a change in the patient’s response. [49] An assessment of the response shift may therefore be needed to obtain a valid and sensitive assessment of change over time. [49] Because the entropy was 0.60 and the mean posterior probability was low (0.67) in the high and declining trajectory (Supplemental Table S5), there was some uncertainty in patient classification.

In conclusion, this study highlights heterogeneity in PCS evolution over time among CKD patients not receiving KRT, with a large sample belonging to the “low stable” or “high declining” PCS group but not the MCS evolution over time. The evolution of specific HRQOL dimensions (symptoms, burden and effect) is superimposed on the evolution of PCS. The decline in eGFR was faster in participants belonging to the “High and declining PCS trajectory” than in those in the other PCS profiles. Patients in the high and stable trajectory had a more favorable clinical and biological profile at baseline. These results contribute to our understanding of Quality-of-life and the importance that monitoring it could have in clinical practice, research and life participation.

## Electronic supplementary material

Below is the link to the electronic supplementary material.

Supplementary Material 1

## Data Availability

The data that support the findings of this study are available upon reasonable request by contacting the CKD-REIN study coordination staff at ckdrein@inserm.fr. Processing of the data supporting the study findings are under the responsibility of the Institut National de la Santé et de la Recherche Medicale (Inserm), France and complies with the European Regulation (EU) 2016/679 (General Data Protection Regulation) related to “the protection of natural persons with regard to the processing of personal data and rules relating to the free movement of personal data”. In compliance with Inserm standard data sharing processes and the agreements obtained from the “Commission nationale de l’informatique et des libertés (CNIL_DR-2012–469)” and the ethics committee (IRB00003888 and CCTIRS12.360/CPP), the data can be made available under request to the study coordinating center via ckdrein@inserm.fr. The code used in the analyses is stored within servers at the Centre de recherche en Epidémiologie et Santé des Population (CESP, Univ Paris-Saclay, Inserm, Villejuif, France) and can also be made available upon request. Any relevant summary statistics for the article are already included within the main article and will be publicly available once the article is published.

## Abbreviations

ACR: Urinary albumin-to-creatinine ratio
AIC: Akaike information criterion
BMI: Body mass index
CES-D: Center for Epidemiologic Studies Depression Scale
CKD: Chronic Kidney Disease
CKD-REIN: Chronic Kidney Disease – Renal Epidemiology Information Network
eGFR: Estimated Glomerular Filtration Rate
GPAQ: Global Physical Activity Questionnaire
HRQOL: Health-related Quality-of-life
JLCMM: Joint Latent Class Mixed Model
KDIGO: Kidney Disease Improving Global Outcomes
KDQOL-36: Kidney Disease Quality of Life − 36
KFRT: Kidney failure with replacement therapy
KRT: kidney replacement therapy
LCMM: Latent class mixed model
MCS: Mental Component Summary
PCS: Physical Component Summary
PROM: Patient-reported Outcome measures
REIN: Renal Epidemiology and Information Network
