# Supplementary material for: Health-related quality of life in nondialysis CKD patients: a comprehensive description of five-year trajectories among the CKD–REIN cohort
Source: BMC Nephrol. 2026 Jan 7;27:90. doi: 10.1186/s12882-025-04702-2 (PMC12869980; doi:10.1186/s12882-025-04702-2)
Supplement: Supplementary file 1 — Supplementary Material 1 [file 12882_2025_4702_MOESM1_ESM.docx]

**SUPPLEMENTAL FILE**

**Supplemental Tables**

**Supplemental Table S1:** STROBE Statement—Checklist of items that should be included in reports of ***cohort studies***. NA = not applicable

|  | Item No | Recommendation | Pages |
| --- | --- | --- | --- |
| **Title and abstract** | 1 | (*a*) Indicate the study’s design with a commonly used term in the title or the abstract | 1-2 |
|  |  | (*b*) Provide in the abstract an informative and balanced summary of what was done and what was found | 2 |
| Introduction | | |  |
| Background/rationale | 2 | Explain the scientific background and rationale for the investigation being reported | 4, lines 2-14 |
| Objectives | 3 | State specific objectives, including any prespecified hypotheses | 4, lines 15-21 |
| Methods | | |  |
| Study design | 4 | Present key elements of study design early in the paper | 5, lines 3-4 |
| Setting | 5 | Describe the setting, locations, and relevant dates, including periods of recruitment, exposure, follow-up, and data collection | 5, lines 6-24 |
| Participants | 6 | (*a*) Give the eligibility criteria, and the sources and methods of selection of participants. Describe methods of follow-up | 5, lines 9-11 |
|  |  | (*b*) For matched studies, give matching criteria and number of exposed and unexposed | NA |
| Variables | 7 | Clearly define all outcomes, exposures, predictors, potential confounders, and effect modifiers. Give diagnostic criteria, if applicable | 5 (lines 21-24); 6 (lines 1-7 |
| Data sources/ measurement | 8* | For each variable of interest, give sources of data and details of methods of assessment (measurement). Describe comparability of assessment methods if there is more than one group | 6 |
| Bias | 9 | Describe any efforts to address potential sources of bias | 6-7 |
| Study size | 10 | Explain how the study size was arrived at | 5 |
| Quantitative variables | 11 | Explain how quantitative variables were handled in the analyses. If applicable, describe which groupings were chosen and why | NA |
| Statistical methods | 12 | (*a*) Describe all statistical methods, including those used to control for confounding | 7 |
|  |  | (*b*) Describe any methods used to examine subgroups and interactions | 6 |
|  |  | (*c*) Explain how missing data were addressed | 6 |
|  |  | (*d*) If applicable, explain how loss to follow-up was addressed | 6 |
|  |  | (*e*) Describe any sensitivity analyses | 8, lines 6-10 |
| Results | | |  |
| Participants | 13* | (a) Report numbers of individuals at each stage of study—eg numbers potentially eligible, examined for eligibility, confirmed eligible, included in the study, completing follow-up, and analysed | 9, Fig. 1 |
|  |  | (b) Give reasons for non-participation at each stage | Fig. 1 |
|  |  | (c) Consider use of a flow diagram | Fig. 1 |
| Descriptive data | 14* | (a) Give characteristics of study participants (eg demographic, clinical, social) and information on exposures and potential confounders | 9, Table 1 |
|  |  | (b) Indicate number of participants with missing data for each variable of interest | 9, Table 1 |
|  |  | (c) Summarise follow-up time (eg, average and total amount) | 9, line 15 |
| Outcome data | 15* | Report numbers of outcome events or summary measures over time | 9, lines 15-16 |
| Main results | 16 | (*a*) Give unadjusted estimates and, if applicable, confounder-adjusted estimates and their precision (eg, 95% confidence interval). Make clear which confounders were adjusted for and why they were included | Table 2 |
|  |  | (*b*) Report category boundaries when continuous variables were categorized | NA |
|  |  | (*c*) If relevant, consider translating estimates of relative risk into absolute risk for a meaningful time period | NA |
| Other analyses | 17 | Report other analyses done—eg analyses of subgroups and interactions, and sensitivity analyses | 11, lines 8-12 |
| Discussion | | |  |
| Key results | 18 | Summarise key results with reference to study Uppobjectives | 12, lines 2-8 |
| Limitations | 19 | Discuss limitations of the study, taking into account sources of potential bias or imprecision. Discuss both direction and magnitude of any potential bias | 16, lines 23-25;  17, lines 1-12 |
| Interpretation | 20 | Give a cautious overall interpretation of results considering objectives, limitations, multiplicity of analyses, results from similar studies, and other relevant evidence | 12-15 |
| Generalisability | 21 | Discuss the generalisability (external validity) of the study results | 16-17 |
| Other information | | |  |
| Funding | 22 | Give the source of funding and the role of the funders for the present study and, if applicable, for the original study on which the present article is based | 20, lines 13-23 |

*Give information separately for exposed and unexposed groups.

**Note:** An Explanation and Elaboration article discusses each checklist item and gives methodological background and published examples of transparent reporting. The STROBE checklist is best used in conjunction with this article (freely available on the Web sites of PLoS Medicine at http://www.plosmedicine.org/, Annals of Internal Medicine at http://www.annals.org/, and Epidemiology at http://www.epidem.com/). Information on the STROBE Initiative is available at http://www.strobe-statement.org.

**Supplemental Table S2**: Definitions of the operational variables used in the study.

| **Variables** | **Definitions** |
| --- | --- |
| Depression score | Assessed by the Center for Epidemiologic Studies Depression Scale (CES-D) depression symptoms index, short Boston form. Variable score standardized on a scale from 0 to 100, with higher scores indicating the presence of more depression. |
| Physical activity | Assessed by the Global Physical Activity Questionnaire (GPAQ) and classified according to WHO physical activity levels:  - High: intense physical activity at least 3 days per week, resulting in an energy expenditure of at least 1500 MET-min/week OR at least 7 days of walking and moderate or intense physical activity until reaching a minimum of 3000 MET-min per week.  - Moderate: not meeting the criteria in the previous category but meeting one of the following criteria: at least 20 min of vigorous physical activity per day for ≥ 3 days per week OR at least 30 min of moderate physical activity or walking per day for ≥ 5 days per week OR at least 5 days of walking and moderate or vigorous physical activity, until a minimum of 600 MET-min per week is achieved.  - Low: not meeting the above criteria. |
| Chronic kidney disease stages (eGFR) | Classification of CKD into 5 stages based on eGFR by Kidney Disease Improving Global Outcomes (KDIGO) 2012. Stages 1 and 2: eGFR ≥ 60 ml/min/1.73 m^2^. Stage 3a: eGFR 45–59 ml/min/1.73 m^2^, Stage 3b: eGFR 30–44 ml/min/1.73 m^2^, Stage 4: eGFR 15–29 ml/min/1.73 m^2^, and Stage 5: eGFR <15 ml/min/1.73 m^2^. eGFR was estimated by the 2009 Chronic Kidney Disease Epidemiology Collaboration equation. |
| Chronic kidney disease stages (ACR) | Classification of CKD according to the ratio of urinary albumin to creatinine (ACR). Category A1: ACR < 30 mg/g, A2: ACR 30 to 299 mg/g, and A3: ACR ≥ 300 mg/g (KDIGO 2012). |
| Dropout | The variables initiating kidney replacement therapy (KRT) and death before KRT were considered dropouts. KRT was defined as the initiation of dialysis (hemodialysis/peritoneal dialysis) or preemptive kidney transplantation. |
| BMI | Body mass index was classified according to the WHO. Underweight: BMI < 18.5 kg/m^2^, healthy weight: 18.5 to <25 kg/m^2^, overweight: 25.0 to <30 kg/m^2^, and obese: ≥ 30.0 kg/m^2^. |
| Diabetes mellitus | Presence of any of the following criteria: 1) treatment with insulin or oral glucose-lowering drugs, 2) fasting blood glucose ≥ 7 mmol/L, 3) random blood glucose ≥ 11 mmol/L, and 4) HbA1c level ≥ 6.5 %. |
| Cardiovascular history | History of stroke, ischemic heart disease or heart failure |
| Anemia | Hemoglobin level < 12.0 g/dl in women and < 13.0 g/dl in men |

**Supplemental Table S3:** Summary table of the statistical criteria for fitting and classification of the different M-class models tested for PCS. The 3-class model with a variance–covariance matrix of the random effects common to all classes was retained. The statistical criteria for several models were not presented because the number of iterations (500 iterations) with or without GridSearch (50 iterations and 100 replications) reached without convergence. Abbreviations: BIC, Bayesian information criterion; npm, number of parameters.

| **Number of classes** | **npm** | **BIC** | **Entropy** | **% Per Class Based on Most Likely Class Membership** | | | |
| --- | --- | --- | --- | --- | --- | --- | --- |
|  |  |  |  | **Class 1** | **Class 2** | **Class 3** | **Class 4** |
| 1 | 18 | 65887.09 | 1.00 | 100 |  |  |  |
| 2^a^ | 29 | 65600.39 | 0.53 | 38.51 | 61.49 |  |  |
| 2^b^ | 30 | 65605.10 | 0.56 | 38.51 | 61.49 |  |  |
| 2^c^ | 29 | 65600.39 | 0.54 | 33.10 | 66.90 |  |  |
| 3^a^ | 37 | 65587.44 | 0.60 | 5.63 | 52.10 | 42.27 |  |
| 3^d^ | 37 | 65648.62 | 0.53 | 19.55 | 79.71 | 0.74 |  |
| 3^b^ | 39 | 65594.06 | 0.60 | 55.34 | 37.08 | 7.58 |  |
| **3^c^** | **37** | **65587.43** | **0.60** | **5.89** | **50.96** | **43.15** |  |
| 3^e^ | 39 | 65595.63 | 0.60 | 55.38 | 36.89 | 7.73 |  |
| 4^b^ | 48 | 65627.09 | 0.53 | 28.09 | 29.86 | 26.77 | 15.28 |
| 4^a^ | 45 | 500 iterations reached without convergence | | | | | |
| 4^d^ | 48 | 500 iterations reached without convergence | | | | | |
| 4^c^ | 45 | 500 iterations reached without convergence | | | | | |
| 4^e^ | 48 | 500 iterations reached without convergence | | | | | |
| 5^a^ | 53 | 500 iterations reached without convergence | | | | | |
| 5^d^ | 53 | 500 iterations reached without convergence | | | | | |
| 5^c^ | 53 | 500 iterations reached without convergence | | | | | |
| ^a^ initial parameters set from 1-class model; ^b^ model with class-specific variance‒covariance matrix of the random effects; ^c^ model with GridSearch (100 replications); ^d^ model with randomization of initials values; ^e^ model with class-specific variance‒covariance matrix of the random effects and GridSearch (100 replications) | | | | | | | |

**Supplemental Table S4:** Summary table of the statistical criteria for fitting and classifying the different M-class models tested for MCS. The 1-class model was retained. The statistical criteria for several models were not presented because the number of iterations (500 iterations) with or without GridSearch (50 iterations and 100 replications) reached without convergence. Abbreviations: BIC, Bayesian information criterion; npm, number of parameters.

| **Number of classes** | **npm** | **BIC** | **Entropy** | **% Per Class Based on Most Likely Class Membership** | | | |
| --- | --- | --- | --- | --- | --- | --- | --- |
|  |  |  |  | **Class 1** | **Class 2** | **Class 3** | **Class 4** |
| **1** | **10** | **63850.05** | **1.00** | **100** |  |  |  |
| 2^a^ | 17 | 63878.21 | 0.80 | 0.63 | 99.37 |  |  |
| 2^b^ | 18 | 63885.11 | 0.86 | 1.03 | 98.97 |  |  |
| 2^c^ | 17 | 63878.21 | 0.80 | 0.63 | 99.37 |  |  |
| 3^a^ | 24 | 63911.63 | 0.66 | 94.62 | 2.87 | 2.50 |  |
| 3^d^ | 24 | 63911.63 | 0.66 | 94.62 | 2.87 | 2.50 |  |
| 3^b^ | 26 | 63928.58 | 0.70 | 94.85 | 2.32 | 2.84 |  |
| 3^c^ | 24 | 63911.62 | 0.66 | 94.59 | 2.54 | 2.87 |  |
| 4^c^ | 31 | 63952.95 | 0.67 | 81.85 | 15.24 | 0.99 | 1.91 |
| 4^a^ | 31 | 500 iterations reached without convergence | | | | | |
| 4^d^ | 31 | 500 iterations reached without convergence | | | | | |
| 5^a^ | 38 | 500 iterations reached without convergence | | | | | |
| 5^d^ | 38 | 500 iterations reached without convergence | | | | | |
| 5^c^ | 38 | 500 iterations reached without convergence | | | | | |
| ^a^ initial parameters set from 1-class model; ^b^ model with class-specific variance‒covariance matrix of the random effects; ^c^ model with GridSearch (100 replications); ^d^ model with randomization of initials values; ^e^ model with class-specific variance‒covariance matrix of the random effects and GridSearch (100 replications) | | | | | | | |

**Supplemental Table S5**: Posterior classification table of the 3-class model for PCS.

| **Class** | **Mean of posterior probabilities in each class** | | |
| --- | --- | --- | --- |
|  | Probability 1 | Probability 2 | Probability 3 |
| Class 1 | **0.6686** | 0.1350 | 0.1964 |
| Class 2 | 0.0220 | **0.8293** | 0.1487 |
| Class 3 | 0.0648 | 0.1169 | **0.8183** |

**Supplemental Table S6:** Comparison of participants included in the analysis (n = 2716) and those excluded (n = 317). CKD–REIN cohort, France.

| **Characteristic** | **Overall** | **Excluded** | **Included** |
| --- | --- | --- | --- |
| Number of participants | 3033 | 317 | 2716 |
| Age (in years) | 66.82 ± 12.87 | 66.22 ± 14.99 | 66.89 ± 12.59 |
| Age group (years) |  |  |  |
| 18–44 | 206 (6.8) | 34 (11) | 172 (6.3) |
| 45–64 | 853 (28) | 81 (26) | 772 (28) |
| 65–74 | 1057 (35) | 95 (30) | 962 (35) |
| ≥ 75 | 917 (30) | 107 (34) | 810 (30) |
| Male sex | 1982 (65) | 190 (60) | 1792 (66) |
| Currently married | 1695 (56) | 37 (12) | 1658 (61) |
| BMI (in kg/m^2^) | 28.71 ± 5.85 | 29.27 ± 6.38 | 28.65 ± 5.78 |
| Body weight status |  |  |  |
| Underweight | 46 (1.5) | 6 (1.9) | 40 (1.5) |
| Healthy weight | 789 (26) | 73 (23) | 716 (26) |
| Overweight | 1082 (36) | 102 (32) | 980 (36) |
| Obesity | 1050 (35) | 123 (39) | 927 (34) |
| Diabetes mellitus | 1307 (43) | 166 (52) | 1141 (42) |
| Cardiovascular history | 1594 (53) | 180 (57) | 1414 (52) |
| Charlson Comorbidity Index ≥ 5 | 2303 (76) | 237 (75) | 2066 (76) |
| Dialysis | 674 (22) | 95 (30) | 579 (21) |
| Kidney transplant | 94 (3.1) | 9 (2.8) | 85 (3.1) |
| Died | 771 (25) | 126 (40) | 645 (24) |
| Number of drugs | 8.04 ± 3.92 | 8.56 ± 3.75 | 7.98 ± 3.94 |
| Burden score | 74.35 ± 24.06 | 56.37 ± 29.17 | 74.83 ± 23.73 |
| Symptoms score | 75.32 ± 16.45 | 68.87 ± 18.98 | 75.50 ± 16.34 |
| Effect of kidney disease score | 81.35 ± 17.91 | 68.05 ± 26.23 | 81.70 ± 17.52 |
| Depression score (CES-D) | 25.24 ± 17.19 | 34.55 ± 19.14 | 25.02 ± 17.09 |
| eGFR (ml/min/1.73 m^2^) | 32.96 ± 12.22 | 30.87 ± 12.67 | 33.21 ± 12.14 |
| CKD stages (eGFR) |  |  |  |
| Stage 2 | 65 (2.1) | 6 (1.9) | 59 (2.2) |
| Stage 3 | 1601 (53) | 145 (46) | 1456 (54) |
| Stage 4 | 1249 (41) | 142 (45) | 1107 (41) |
| Stage 5 | 118 (3.9) | 24 (7.6) | 94 (3.5) |
| eGFR < 30 mL/min/1.73 m^2^ | 1367 (45) | 166 (52) | 1201 (44) |
| ACR (mg/g) |  |  |  |
| < 30 | 767 (25) | 70 (22) | 697 (26) |
| 30 to 300 | 861 (28) | 79 (25) | 782 (29) |
| > 300 | 1130 (37) | 128 (40) | 1002 (37) |
| Anemia | 1143 (38) | 138 (44) | 1005 (37) |
| Serum albumin < 40 g/L | 245 (8.1) | 40 (13) | 205 (7.5) |
| All data are presented as n (%) or mean ± SD.  Abbreviations: ACR, urinary albumin‒creatinine ratio; BMI, body mass index; eGFR, estimated glomerular filtration rate; MCS, mental component summary; PCS, physical component summary; CKD, chronic kidney disease; KRT, kidney replacement therapy; GPAQ, Global Physical Activity Questionnaire; CES-D, Center for Epidemiologic Studies Depression Scale  A higher score indicates the presence of more depression or best quality of life (PCS, MCS, burden, and effect) | | | |

**Supplemental Table S7:** Summary table of the statistical criteria for fitting and classification of the different M-class models tested for physical component summary by *lcmm* function of the LCMM R package. The 2-class model with a variance–covariance matrix of the random effects common to all classes was retained. The statistical criteria for several models were not presented because the number of iterations (500 iterations) with or without GridSearch (50 iterations and 100 replications) reached without convergence.

| **Number of classes** | **npm** | **BIC** | **Entropy** | **% Per Class Based on Most Likely Class Membership** | | | | |
| --- | --- | --- | --- | --- | --- | --- | --- | --- |
|  |  |  |  | **Class 1** | **Class 2** | **Class 3** | **Class 4** | **Class 5** |
| 1 | 17 | 56668.04 | 1.00 | 100 |  |  |  |  |
| 2^a^ | 21 | 56676.24 | 0.41 | 59.32 | 40.68 |  |  |  |
| 2^b^ | 21 | 56663.93 | 0.98 | 0.22 | 99.78 |  |  |  |
| **2^c^** | **22** | **56647.00** | **0.78** | **7.44** | **92.56** |  |  |  |
| **2^d^** | **22** | **56647.00** | **0.78** | **7.44** | **92.56** |  |  |  |
| 3^a^ | 25 | 56677.41 | 0.59 | 0.63 | 39.06 | 60.31 |  |  |
| 3^b^ | 25 | 56677.41 | 0.59 | 0.63 | 39.06 | 60.30 |  |  |
| 3^c^ | 27 | 56662.77 | 0.73 | 9.54 | 89.36 | 1.10 |  |  |
| 3^d^ | 27 | 56655.26 | 0.84 | 92.67 | 7.03 | 0.29 |  |  |
| 4^a^ | 29 | 500 iterations reached without convergence | | | | | |  |
| 4^b^ | 29 | 56679.43 | 0.66 | 57.51 | 0.37 | 14.06 | 28.06 |  |
| 4^c^ | 32 | 56662.36 | 0.53 | 55.96 | 26.14 | 3.68 | 14.21 |  |
| 4^d^ | 32 | 56660.60 | 0.76 | 89.76 | 0.52 | 8.36 | 1.36 |  |
| 5^a^ | 33 | 500 iterations reached without convergence | | | | | |  |
| 5^b^ | 33 | 500 iterations reached without convergence | | | | | |  |
| 5^c^ | 37 | 56688.50 | 0.56 | 1.77 | 0.81 | 62.22 | 5.19 | 30.01 |
| 5^d^ | 37 | 56677.31 | 0.63 | 1.47 | 52.87 | 0.81 | 28.31 | 16.53 |
| ^a^ initial parameters set from 1-class model; ^b^ model with GridSearch (100 replications); ^c^ model with class-specific variance‒covariance matrix of the random effects; ^d^ model with class-specific variance‒covariance matrix of the random effects and GridSearch (100 replications).  BIC, Bayesian Information Criterion; npm, number of parameters. | | | | | | | | |

**Supplemental Table S8:** Summary table of the statistical criteria for fitting and classification of the different M-class models tested for mental component summary score by *lcmm* function of the LCMM R package. The 1-class model was retained. The statistical criteria for several models were not presented because the number of iterations (500 iterations) with or without GridSearch (50 iterations and 100 replications) reached without convergence.

| **Number of classes** | **npm** | **BIC** | **Entropy** | **% Per Class Based on Most Likely Class Membership** | | | | |
| --- | --- | --- | --- | --- | --- | --- | --- | --- |
|  |  |  |  | **Class 1** | **Class 2** | **Class 3** | **Class 4** | **Class 5** |
| **1** | **6** | **54631.53** | **1.00** | **100** |  |  |  |  |
| 2^a^ | 9 | 54655.25 | 0.00 | 42.42 | 57.58 |  |  |  |
| 2^b^ | 9 | 54634.96 | 0.94 | 0.29 | 99.7 |  |  |  |
| 2^c^ | 10 | 54643.14 | 0.90 | 99.63 | 0.37 |  |  |  |
| 2^d^ | 10 | 54643.14 | 0.90 | 0.37 | 99.63 |  |  |  |
| 3^a^ | 12 | 54658.68 | 0.76 | 0.00 | 99.71 | 0.29 |  |  |
| 3^b^ | 12 | 54651.60 | 0.88 | 0.29 | 99.37 | 0.33 |  |  |
| 3^c^ | 14 | 54664.72 | 0.80 | 99.23 | 0.33 | 0.44 |  |  |
| 3^d^ | 14 | 54664.72 | 0.80 | 99.23 | 0.33 | 0.44 |  |  |
| 4^a^ | 15 | 54671.40 | 0.59 | 26.95 | 0.33 | 72.64 | 0.07 |  |
| 4^b^ | 15 | 54671.40 | 0.59 | 26.51 | 0.33 | 73.09 | 0.07 |  |
| 4^c^ | 18 | 54692.91 | 0.74 | 98.67 | 0.99 | 0.00 | 0.33 |  |
| 4^d^ | 18 | 54691.82 | 0.75 | 98.67 | 0.00 | 0.33 | 0.99 |  |
| 5^a^ | 18 | 500 iterations reached without convergence | | | | | |  |
| 5^b^ | 18 | 500 iterations reached without convergence | | | | | | |
| 5^c^ | 22 | 500 iterations reached without convergence | | | | | | |
| ^a^ initial parameters set from 1-class model; ^b^ model with GridSearch (100 replications); ^c^ model with class-specific variance‒covariance matrix of the random effects; ^d^ model with class-specific variance‒covariance matrix of the random effects and GridSearch (100 replications). BIC, Bayesian Information Criterion; npm, number of parameters. | | | | | | | | |

**Supplemental Figures**


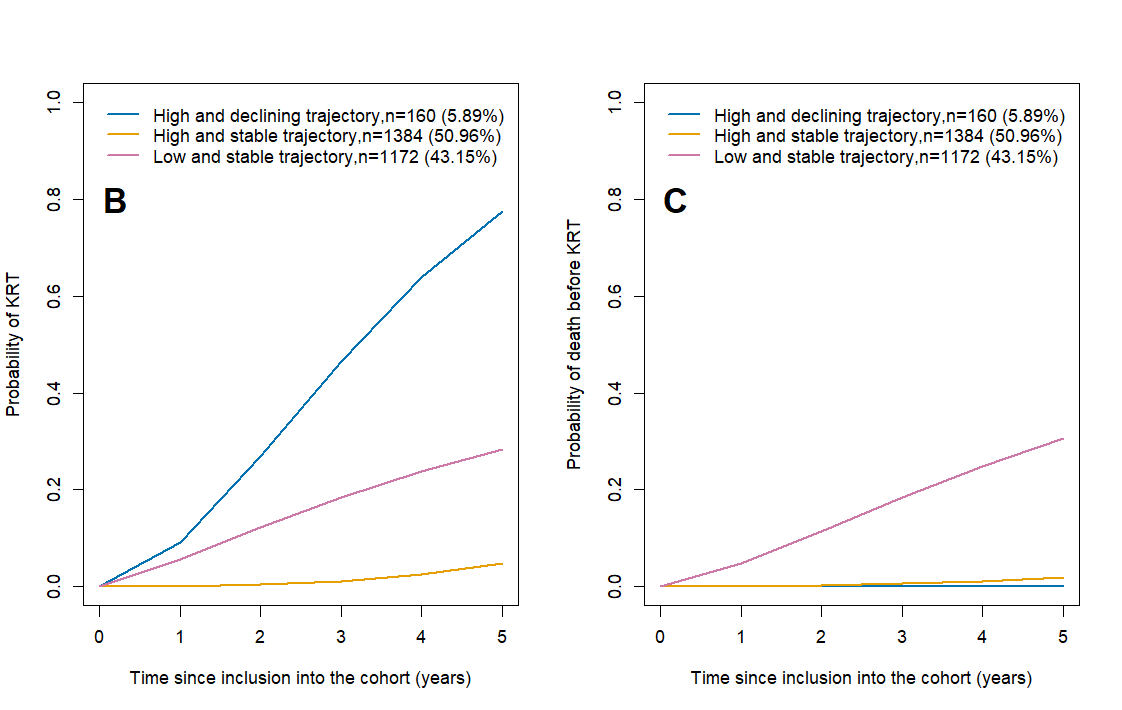


**Supplemental Figure S1**: Outcome’s probability according to PCS trajectories. The panel **B** shows the crude probability of kidney failure with replacement therapy (KFRT) by PCS class trajectories. The panel **C** shows the crude probability of death before KFRT by PCS class trajectories.


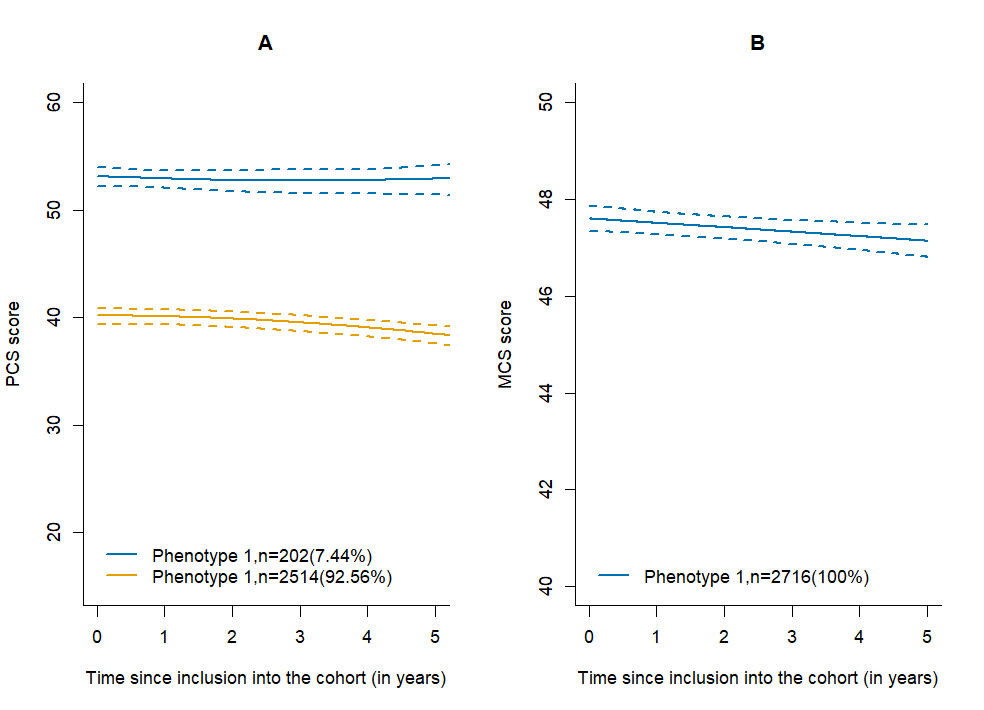


**Supplemental Figure S2**: Predicted trajectories of physical and mental HRQOL.

The panel **A** shows the predicted mean trajectories (solid line) of physical HRQOL in the two identified latent classes by *lcmm* function and their confidence intervals (dotted line). The panels **B** shows the predicted mean trajectory (solid line) of mental HRQOL identified by *lcmm* function and their confidence intervals (dotted line). The lower the HRQOL score is, the worse the HRQOL.

**Supplemental Text**

**Supplemental Text S1**: Main analysis strategies

Modeling the best link function was the first step of the main analysis. For mental HRQoL, the linear link function was used because the distribution of scores follows a normal distribution. For physical HRQoL, a joint 1-class unadjusted model with no covariates other than time was used to test the best link function between the observed scores and the latent process because the distribution of scores does not follow a normal distribution. We tested the following link functions: linear, cumulative distribution function of the beta distribution (beta-CDF), natural splines with three knots at quantiles, natural splines with five knots at quantiles, natural splines with seven knots at quantiles, natural splines with three equidistant knots and natural splines with five equidistant knots. These seven models were compared with the Akaike information criterion (AIC) and number of parameters. The link function with the smallest AIC was selected. This step allowed us to retain the natural splines link function with seven knots at quantiles. Thus, the natural splines link function with seven knots at quantiles was used in all other models of physical HRQoL, and the linear function was used in models of mental HRQoL.

In the second step, several combinations of risk (survival model) and time (mixed model) functions with an unstructured or diagonal variance‒covariance matrix (idiag = TRUE or FALSE) were tested. Twenty models (20 combinations) with 1 class were tested and compared in terms of the AIC and number of parameters for assessing physical and mental HRQoL. This step resulted in the choice of the model combining a natural spline with one interior knot at the median (for the time function) and a Weibull hazard function for the two events with an unstructured variance‒covariance matrix for physical HRQoL and a linear time function and a Weibull hazard function for the two events with an unstructured variance‒covariance matrix for mental HRQoL.

Using these functions and an unstructured variance‒covariance matrix, we tested one- to five-class empty models other than time with and without class-specific variance‒covariance matrix of the random effects by using the following strategy:

- First, we fitted a one-class model with the structure retained in the previous step of the analysis.

> Script code R example for 1-class JLCMM for physical HRQoL

Mod1 = jlcmm(PCS~ ns(time, knots = 1.3907, Boundary.knots = c(0,5.7123)),

random = ~ ns(time, knots = 1.3907, Boundary.knots = c(0,5.7123)),

survival = surv(Tevent, event)~cause1(1)+cause2(1),

hazard = c("Weibull","Weibull"),

maxiter = 200,

link = "7-quant-splines",

subject = "id_ckdrein",

data = base_lcmm)

- We systematically set the initial parameters from the 1-class model (mod1). This was achieved by specifying the number of classes (‘ng = M’) and the initial parameters (‘B = mod1’) to the ‘jlcmm’ function.

> Script code R example for 3-class JLCMM

pcs3_jlcmm = jlcmm(PCS~ns(time, knots = 1.3907, Boundary.knots = c(0,5.7123)),

random = ~ ns(time, knots = 1.3907, Boundary.knots = c(0,5.7123)),

mixture = ~ns(time, knots = 1.3907, Boundary.knots = c(0,5.7123)),

survival = surv(Tevent, event)~cause1(1)+cause2(1),

**B = mod1**,

link = "7-quant-splines",

subject = "id_ckdrein",

**ng = 3**,

data = base_lcmm,

maxiter = 500)

- If the M-class model did not converge after 500 iterations, we substituted the estimates from the M-class LCMM that converge into the vector of parameter estimates from the M-class joint model and then refit the model using those parameter estimates as starting values for a new attempt at fitting. This was achieved by specifying the initial parameters (‘B = inits’) to the ‘jlcmm’ function with ‘inits’ being a randomly drawn set of initial values.

> Script code R example for fitting a latent class joint model with 4 classes, using a randomly drawn set of initial values

# Function to draw initial values for 4 classes

val_inits_4 <- function() {

c(

intercept_class1 = -3.0276779,

intercept_class2 = 0.2534492,

intercept_class3 = -3.4329952,

event1_weibull1_class1 = rnorm(1, 0, 1),

event1_weibull2_class1 = rnorm(1, 0, 1),

event1_weibull1_class2 = rnorm(1, 0, 1),

event1_weibull2_class2 = rnorm(1, 0, 1),

event1_weibull1_class3 = rnorm(1, 0, 1),

event1_weibull2_class3 = rnorm(1, 0, 1),

event1_weibull1_class4 = rnorm(1, 0, 1),

event1_weibull2_class4 = rnorm(1, 0, 1),

event2_weibull1_class1 = rnorm(1, 0, 1),

event2_weibull2_class1 = rnorm(1, 0, 10),

event2_weibull1_class2 = rnorm(1, 0, 1),

event2_weibull2_class2 = rnorm(1, 0, 1),

event2_weibull1_class3 = rnorm(1, 0, 1),

event2_weibull2_class3 = rnorm(1, 0, 1),

event2_weibull1_class4 = rnorm(1, 0, 1),

event2_weibull2_class4 = rnorm(1, 0, 1),

intercept_class2 = -0.5068667,

intercept_class3 = 2.2369242,

intercept_class4 = 1.9615968,

ns1_class1 = 2.3773060,

ns1_class2 = -0.3909669,

ns1_class3 = -5.6179982,

ns1_class4 = -0.2546055,

ns2_class1 = -6.0258363,

ns2_class2 = -0.6034931,

ns2_class3 = 6.3377442,

ns2_class4 = -0.1591931,

varcov1 = 1.7367845,

varcov2 = -0.7750556,

varcov3 = 2.1347513,

varcov4 = -0.6119836,

varcov5 = 0.9012272,

varcov6 = 0.4362015,

I_splines1 = -7.1052402,

I_splines2 = 1.6905537,

I_splines3 = 1.3837831,

I_splines4 = 1.3977475,

I_splines5 = 1.0716104,

I_splines6 = 0.9056008,

I_splines7 = 1.3122338,

I_splines8 = 1.8063767,

I_splines9 = 0.6580010

)

}

seed <- rint() # generate a random seed

set.seed(seed) # set the seed for reproducibility

inits4 <- val_inits_4() # randomly draw a set of initial values

- A grid search with 100 replications and 50 iterations was systematically performed for the models with more than one class.

> Script code R example for 3-class JLCMM with grid search

grid_pcs3_jlcmm <- gridsearch(rep = 100, maxiter = 50, minit = mod1,

jlcmm(PCS~ns(time, knots = 1.3907, Boundary.knots = c(0,5.7123)),

random = ~ ns(time, knots = 1.3907, Boundary.knots = c(0,5.7123)),

mixture = ~ns(time, knots = 1.3907, Boundary.knots = c(0,5.7123)),

survival = surv(Tevent, event)~cause1(1)+cause2(1),

link = "7-quant-splines",

subject = "id_ckdrein",

ng = 3,

data = base_lcmm,

maxiter = 500))

- For each model, we systematically tested a variance covariance matrix common to the classes (nwg = FALSE) and then specific to each class (nwg = TRUE).
- All these models were systematically rerun several times to avoid model convergence to a local maximum likelihood. The 3-class model for the physical HRQoL in particular was rerun approximately twenty times with or without grid search.
